# Interleukin-25 Induces Resistance Against Intestinal Trematodes

**DOI:** 10.1038/srep34142

**Published:** 2016-09-23

**Authors:** Carla Muñoz-Antoli, Alba Cortés, Rebeca Santano, Javier Sotillo, J. Guillermo Esteban, Rafael Toledo

**Affiliations:** 1 Departamento de Parasitología, Facultad de Farmacia, Universidad de Valencia, Av. Vicente Andrés Estellés s/n, 46100 Burjassot - Valencia, Spain

## Abstract

*Echinostoma caproni* is an intestinal trematode that has been extensively used as an experimental model to investigate the factors determining the resistance to intestinal helminths or the development of chronic infections. ICR mice are permissive hosts for *E. caproni* in which chronic infections are developed, concomitantly with local Th1 responses, elevated levels of local IFN-γ, inflammation and antibody responses. However, mice develop partial resistance to homologous challenge infections after cure of a primary infection, which converts this subject into an adequate model for the study of the mechanisms generating resistance against intestinal helminths. The purpose of the present study was to compare the immune response induced in primary and secondary infections to elucidate the factors determining the different outcome of the infection in each type of infection. The results obtained indicate that susceptibility is determined by the lack of IL-25 expression in response to primary infection. In contrast, infection in an environment with elevated levels of IL-25, as occurs in challenge infection, results in a Th2 phenotype impairing parasite survival. This was confirmed by treatment of naïve mice with exogenous IL-25 and subsequent infection. Changes induced in goblet cell populations and mucin glycosylation could be implicated in resistance to infection.

Intestinal helminth infections are among the most prevalent infections and it is estimated that more than 1.5 billion people are currently infected with one or more species of intestinal helminth mainly in Asia, Africa and Latin-America^1^,^2^. Notably, children experience the highest prevalences and intensities of infection in these areas with over 270 million preschool-age and over 600 million school-age children currently infected^2^. An important number of these infections are caused by intestinal trematodes with estimates reaching 40 million people infected and more than 10% of the world’s population at risk of infection^3^,^4^,^5^,^6^. Commonly, intestinal helminths cause chronic and debilitating diseases associated with diarrhea, abdominal pain, malabsorption syndrome, vitamin deficiencies, anemia, growth retardation and/or impaired cognitive function among other symptoms^2^,^7^,^8^,^9^. Additionally, intestinal helminths cause substantial economic losses in livestock production worldwide^10^,^11^.

Although treatment of intestinal helminth infections is in general effective, it is expensive and difficult to implement. Moreover, reinfections are continuous and resistant strains are emerging^1^. In this context, there is urgent need for expanding research activity on developing more effective interventions such as vaccination. However, a more comprehensive understanding of the parasite’s biology and the host-parasite interactions of these infections is required. In this context, the use of different host-parasite experimental models has been proven to be a useful approach to investigate the factors determining the resistance to intestinal helminths.

Echinostomes are common human parasites in East and Southeast Asia^12^. Moreover, echinostomes, and particularly *Echinostoma caproni* (Trematoda: Echinostomatidae), have been extensively used for the study the factors on which rely the establishment of chronic infections or, in contrast, the development of resistance to intestinal helminths. *E. caproni* is an intestinal trematode with no tissue phase in the vertebrate definitive host^13^. After infection, the metacercariae excyst in the duodenum and the juvenile worms migrate to the ileum, where they attach to the mucosa. *E. caproni* has a wide range of definitive hosts, although its compatibility differs considerably between rodent species in terms of worm survival and development^14^. In mice and other hosts of high compatibility, the infection becomes chronic, while in hosts of low compatibility, (e.g. rats) the worms are expelled from the 2–4 weeks post-infection^15^,^16^. Moreover, the consequences of the infection in each host class are markedly different. The establishment of chronic infections in ICR mice is dependent upon a local Th1 response with elevated production of IFN-γ^17^. The infection induces important inflammatory responses, a marked epithelial injury and a rapid increase of iNOS expression^16^,^17^. In contrast, the resistance to *E. caproni* infection in hosts of low compatibility is associated with the development of a local Th2/Th17 phenotype and changes in tissue structure are not observed^17^,^18^. Because of these characteristics, the *E. caproni*-rodent model is extensively used to elucidate several aspects of the host-parasite relationships in intestinal infections, such as the induction of distinct effector mechanisms and their effectiveness in parasite clearance.

Although mice are permissive host for *E. caproni*^14^,^19^, a recent study showed that primary infection in ICR mice induces acquired immunity against subsequent homologous infection which is manifested in reduced infection rate, worm recovery and growth^20^. This makes the *E. caproni*/ICR mouse system an excellent model for the study of the mechanisms generating acquired immunity against intestinal helminths. Herein, we use the *E. caproni*/mice system to analyze the regulation of immune responses against intestinal helminths and the factors governing the resistance against these parasites. To this purpose, we followed two approaches. We have extended previous studies on the responses associated to chronicity (primary infections) and compared these responses to those related to resistance to infection (secondary infections). In addition, we have also analyzed other immune mechanisms induced by primary response that have been associated with resistance against intestinal helminth infections.

## Results

### Mice develop resistance against *E. caproni* in secondary infections and after treatment with rIL-25

Mice in infected, praziquantel (pzq)-treated and reinfected groups became infected after primary exposure to *E. caproni* metacercariae. In secondary infections, however, rates of infection were significantly lower than those observed in primary infections (*p* < 0.001). Only 54% of mice belonging to the reinfected group became infected after secondary exposure to *E. caproni* metacercariae. Similarly, worm recoveries were lower in secondary than in primary infections (*p* < 0.001). In primary infections, worm recoveries at 2 and 4 weeks post-primary infection (wppi) ranged from 54 to 94% (76.0 ± 16.6), whereas they were between 0 and 63% (29.9 ± 26.8) at 2 and 4 weeks post-secondary infection (wpsi). Strikingly, mice treated with rIL-25 were refractory to primary infection with 50 metacercariae of *E. caproni* and all the animals were negative at 3, 7 and 14 days post-primary infection.

### Different cytokine profile is associated with susceptibility or resistance to infection

**Figure 1:**
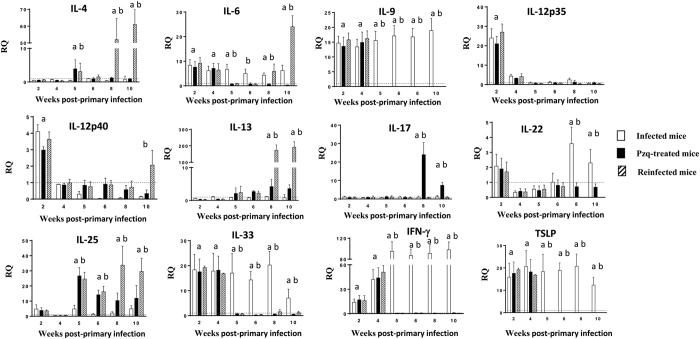
Different cytokine profile is associated with primary and secondary infections. Expression of cytokine mRNA in the intestinal tissue of ICR mice infected, praziquantel (pzq)-treated and reinfected with *Echinostoma caproni*. The relative quantities (RQ) of cytokine genes are shown after normalization with β-actin and standardization of the relative amount against day 0 sample. Vertical bars represent the standard deviation. a: significant differences with respect to negative controls; b: significant differences between groups at each week of the study (p < 0.05).

Changes in cytokine expression as a consequence of primary infection, drug cure and secondary infection were investigated by real-time PCR. The expression of almost all the cytokines studied showed alterations over the course of the experiment (Fig. 1). Only IL-5, IL-10, IL-23A and TGF-β remained unchanged in our study (see Supplementary Figure 1).

Primary infection was characterized by significant elevations of IL-6, IL-9, IL-12p35, IL-12p40, IL-33, IFN-γ and TSLP. However, the values of IL-12p35 and IL-12p40 decreased at 4 wppi in the three groups of mice. In the group of infected animals, the values of IL-6, IL-9, IL-33, IFN-γ and TSLP remained elevated until the end of the experiment (Fig. 1).

Drug cure resulted in a sudden decrease of most of the cytokines that became overexpressed after primary infection (IL-6, IL-9, IL-12, IL-33, IFN-γ and TSLP). However, worm elimination induced an increase in the expression of IL-25 as observed both in the pzq-treated and reinfected groups from 5 wppi. In both groups the levels of IL-25 remained elevated until the end of the experiment (Fig. 1). Secondary infection induced a rapid increase in the expression of IL-4, IL-6 and IL-13 from 2 wpsi and beyond. The expression of the remainding cytokines, except IL-25, remained at low level (Fig. 1).

**Figure 2:**
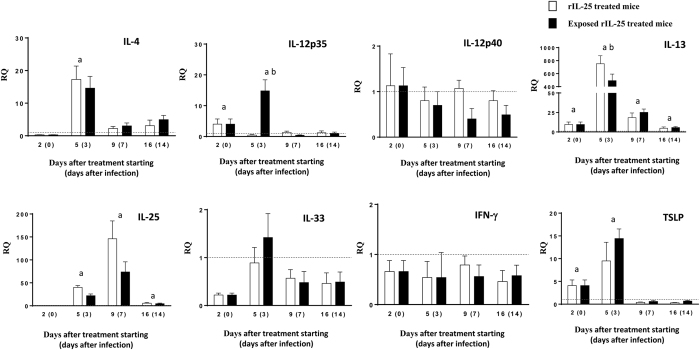
Treatment with rIL-25 induces a Th2 phenotype concomitantly with resistance to infection. Expression of cytokine mRNA in the intestinal tissue of rIL-25-treated ICR mice and rIL-25-treated and infected ICR mice with *Echinostoma caproni*. The relative quantities (RQ) of cytokine genes are shown after normalization with β-actin and standardization of the relative amount against day 0 sample. Vertical bars represent the standard deviation. a: significant differences with respect to negative controls; b: significant differences between groups at each week of the study (p < 0.05).

The levels of cytokines in rIL-25-treated mice are shown in Fig. 2. Treatment of mice with exogenous IL-25 induced elevated levels of IL-4 and, mainly, IL-13. After exposure to *E. caproni* metacercariae, the values of these cytokines progressively decreased, though they remained significantly higher than in control animals until the end of the experiment. As occurred in primarily infected mice, an early and transient upregulation of IL-12p35 was observed, though the values rapidly returned to normal levels. Interestingly, exogenous IL-25 induced marked overexpression of endogenous IL-25 that remained positive during the complete experiment. In contrast, IL-33 and IFN-γ were not affected by treatment with IL-25.

### Primary and secondary infection induces differential activation of macrophages

**Figure 3:**
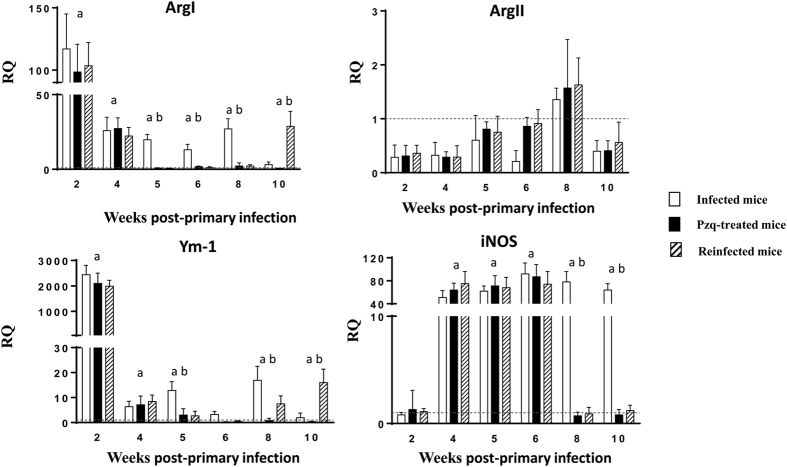
Pattern of macrophage activation is different in primary and secondary infections. Expression of markers mRNA of both classical (Arg2 and iNOS) and alternative (ArgI and Ym-1) activation of macrophages in the intestinal tissue of ICR mice infected, praziquantel (pzq)-treated and reinfected with *Echinostoma caproni*. The relative quantities (RQ) of cytokine genes are shown after normalization with β-actin and standardization of the relative amount against day 0 sample. Vertical bars represent the standard deviation. a: significant differences with respect to negative controls; b: significant differences between groups at each week of the study (p < 0.05).

To study the role of macrophages in the course of *E. caproni* primary and secondary infections and the pathology induced by the parasite, we have analyzed several markers of classical or M1 (ArgII and iNOS) or alternative or M2 (ArgI and Ym-1) activation of macrophages. Interestingly, we found that primary infection with *E. caproni* simultaneously induced overexpression of markers of M1 (iNOS) and M2 (ArgI and Ym-1) activation (Fig. 3). In the group of infected animals, expression levels remained significantly higher than in control animals in the most of the wppi studied, mainly in the case of iNOS. After drug cure, the levels of ArgI and YM-1 returned to normal values and remained at this level in the group of treated animals until the end of the experiment. The expression of iNOS decreased to normal values from 8 wppi in all the animals treated with pzq. Secondary infection resulted in a new increase of ArgI and Ym-1 expression, whereas the expression of iNOS was similar to control animals.

### Partial and complete resistance is associated with goblet cell expansion

**Figure 4:**
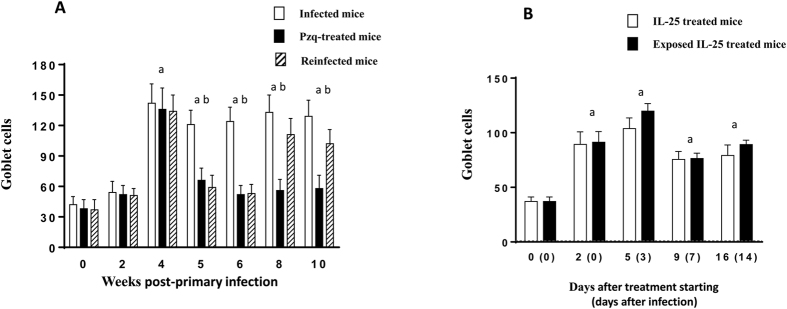
Infection and treatment with rIL-25 induce goblet cell expansion. Kinetics of goblet cells in (**A**) mice infected, praziquantel (pzq)-treated and reinfected with *Echinostoma caproni*. and (**B**) rIL-25-treated ICR mice and rIL-25-treated and infected ICR mice with *Echinostoma caproni*. Vertical bars represent the standard deviation. a: significant differences with respect to negative controls; b: significant differences between groups at each week of the study (p < 0.05).

Primary *E. caproni* infections induced a marked goblet cell expansion from 4 wppi, reaching values of about 3-fold higher than in control animals. Thereafter, the counts remained elevated until the end of the experiment. Drug cure of the infection resulted in a sudden decrease of goblet cell counts, returning to normal values at 6 wppi. Secondary infection induced a rapid goblet cell hyperplasia at 8 wppi (2 wpsi) reaching similar values than in primary infections (Fig. 4A).

Treatment of naïve mice with rIL-25 induced rapid goblet cell expansion (about 3-fold higher than in control animals) detectable from 2 days post-treatment with rIL-25. Goblet cell hyperplasia was constant over the complete course of the experiment. Infection of rIL-25-treated mice did not induce changes in goblet cell counts with respect to non-infected rIL-25-treated mice (Fig. 4B).

### Both *E. caproni* primary and secondary infection induce tuft cell expansion

**Figure 5:**
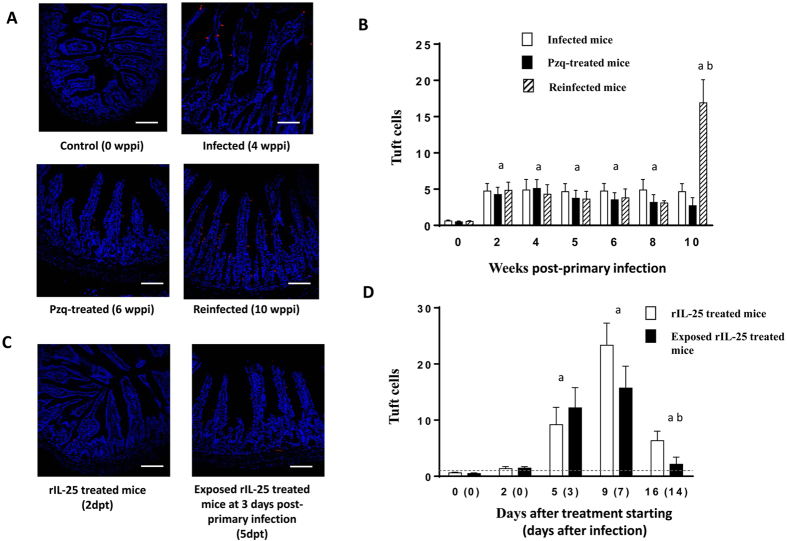
Tuft cell hyperplasia is induced by both infection and treatment with rIL-25. Kinetics of tuft cells in (**A**,**B**) mice infected, praziquantel (pzq)-treated and healed from a primary infection and secondarily infected with *Echinostoma caproni* and (**C**,**D**) rIL-25-treated ICR mice and rIL-25-treated and infected ICR mice with *Echinostoma caproni*. Vertical bars represent the standard deviation. a: significant differences with respect to negative controls; b: significant differences between groups at each week of the study (p < 0.05). wppi: weeks post-primary infection; dpt: days after infection. Scale bar: 30 μm.

As tuft cells may play a major role in resistance against intestinal helminths, we investigated tuft cell responses in each group of mice (Fig. 5). Primary *E. caproni* infection induced a rapid tuft cell expansion of about 8-fold greater than naïve animals (Fig. 5A,B). Tuft cell hyperplasia was detectable from 2 wppi, and the values remained at this level until the end of the experiment. The extent of this hyperplasia was uniform throughout the ileum. Drug cure of primary infection did not affect the tuft cell counts, which remained elevated. In contrast, secondary infection elicited a dramatic (>30-fold higher with respect to negative controls) tuft cell hyperplasia at 2 wpsi (Fig. 5B).

Treatment of naïve mice with exogenous IL-25 also induced expansion of tuft cells (of about 37-fold greater than naïve mice) that peaked at the 9^th^ day after starting the treatment with rIL-25. Exposure to metacercariae of rIL-25-treated mice did not alter tuft cell counts that were similar to those observed in non-infected rIL-25-treated mice (Fig. 5C,D).

### Infection with *E. caproni* and treatment with rIL-25 induce changes in glycosylation of mucins

**Figure 6:**
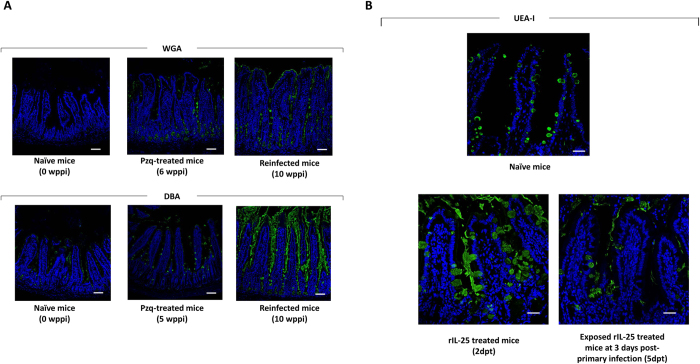
Infection and treatment with rIL-25 induce changes in mucin glycosylation. Images of fluorescent microscopy of ileal sections from (**A**) mice infected, praziquantel (pzq)-treated and reinfected with *Echinostoma caproni* and (**C**,**D**) rIL-25-treated ICR mice and rIL-25-treated and infected ICR mice with *Echinostoma caproni* stained with WGA, DBA and UEA-I lectins. wppi: weeks post-primary infection; dpt: days after infection. Scale bar: 30 μm.

To study alterations in the saccharide residues of mucins induced by *E. caproni* infections and rIL-25 treatment which can be associated with resistance development, four lectins with different ligand specificity were used to stain the ileal mucosa of mice belonging to the different groups. We have focused our analysis on the specific staining of brush border (transmembrane mucins), extracellular mucus (gel-forming mucins) and goblet cell vesicles (mucus producing cells) (Tables 1 and 2; Fig. 6).

Specific staining was undetected using WGA lectin in control animals. After primary infection, a slight increase in the intensity of staining was observed. The intensity of staining increased further after drug cure and reinfection. (Table 1; Fig. 6). Using DBA lectin, specific staining was detected in control animals. As a consequence of primary infection, a slight increase was observed at 4 wppi, though drug cure induced a drastic decrease. The staining intensity became higher after secondary infection at 10 wppi (4 wpsi). Staining of mucus and goblet cell vesicles in naïve mice was detected using UEA-I. A few changes were observed after primary infection and a slight decrease was observed after secondary infection (Table 1; Fig. 6). Using SNA lectin, no significant changes were observed during the experiment.

Treatment of naïve mice with exogenous IL-25 only induced slight changes as compared with control animals using WGA, DBA and SNA lectins (Table 2). Moreover, infection of rIL-25-treated mice did not induce significant changes with respect to naïve mice. In contrast, relevant changes were observed when using UEA-I lectin. A marked increase of specific staining of mucus and goblet cell vesicles was detected from 2 days post-treatment with rIL-25 and the intensity remained elevated until the end of the experiment. Strikingly, exposure to *E. caproni* metacercariae induced a significant decrease in the intensity of staining with this lectin (Table 2; Fig. 6).

## Discussion

Contrary to what occurs with most intestinal helminths, *E. caproni* primary infections in ICR mice induce a local Th1 response with increased expression of IFN-γ^17^. This response is associated with the development of chronic infections with elevated inflammation and tissue damage^16^. However, mice become resistant to homologous secondary infections after drug cure of a primary infection^20^,^21^. Due to these characteristics, we have used the *E. caproni*-mouse system to analyze the factors governing the regulation of immune responses against intestinal helminths and their consequences on the outcome of the infection. Our results support IL-25 induces resistance to *E. caproni* infection and that susceptibility to primary infection relies on the the inability of mice to express IL-25 in response to infection. In contrast, resistance to secondary infection appears to be related to the boost of IL-25 induced by the cure of the infection. These features indicate that IL-25, but not IL-33 and/or TSLP, is essential for resistance to infection.

Primary *E. caproni* infection induced a mucosal environment consistent with a local Th1 response as described previously^17^. However, this response is much more complex than previously thought. Concomitantly with elevated levels of Th1 indicators (IL-12, IFN-γ and iNOS), primary infections induced increased expression of several Th2 markers (IL-33, TSLP, ArgI and Ym-I) and other cytokines such as IL-9. IL-33 and TSLP are alarmins produced by epithelial cells in response to injury and, together with IL-25, act on other innate cells (type 2 innate lymphoid cells or ILC2) to produce IL-4 and/or IL-13 promoting Th2 responses^22^,^23^,^24^,^25^. The increased expression of IL-33 and TSLP in primary *E. caproni* infections in mice could be expected due to the severe tissue damage induced^20^,^21^. However, the lack of IL-25 overexpression was striking, especially considering that infection induced a marked tuft cell hyperplasia. Tuft cells constitutively express IL-25 and, after helminth infection, tuft cell-derived IL-25 activates ILC2 to secrete IL-13 that, via IL-4Rα signaling induces tuft cell hyperplasia and increased levels of IL-25 in a feed-forward circuit promoting Th2 responses against helminths^26^,^27^,^28^. *E. caproni* primary infection induced a tuft cell expansion of about 8-fold which is similar to that induced by other intestinal helminths such as *Nippostrongylus brasiliensis, Heligmosomoides polygyrus* or *Trichinella spiralis*^26^,^27^. However, the expansion of the tuft cell lineage was not accompanied by an increase in IL-25 expression in *E. caproni* primary infection. Probably, parasite-induced signals do not enhance IL-25 production by tuft cells or disrupt transduction signal pathways in these cells. These topics need to be explored further to obtain a better understanding on how parasites can evade host responses.

In the absence of IL-25, the early production of both subunits of IL-12 (IL-12p35 and IL-12p40) may be essential for the determination of the susceptibility to infection. IL-12 (or IL-12p70) is a key pro-inflammatory cytokine that promotes the differentiation toward a Th1 phenotype and stimulates IFN-γ production inhibiting the expression of IL-13^29^,^30^. Under these conditions, primary infections resulted in the development of a local Th1 phenotype as shown by upregulation of IFN-γ and low levels of IL-4 and IL-13. This suggests that, in absence of IL-25, the alarmins IL-33 and TSLP are not sufficient to facilitate the development of protective Th2 responses in the *E. caproni* primary infections in ICR mice.

To confirm the crucial role of tuft cells and IL-25 in the generation of Th2 responses in *E. caproni* infections, mice were treated with rIL-25. All rIL-25-treated mice were refractory to infection. Exogenous IL-25 induced tuft cell expansion together with a strongly polarized Th2 environment with elevated levels of IL-13. It is well known that IL-25 inhibits Th1 differentiation facilitating Th2 differentiation and protecting against intestinal helminth infections^31^,^32^. Similarly to our results, absence of innate IL-25 expression facilitate chronicity of *H. polygyrus* in mice^32^. Under these conditions, rIL-25-treated mice were resistant to infection as determined by necropsy from 3 days post-primary infection (dppi). The levels of IL-33 and TSLP were not affected by IL-25 treatment, confirming the major role of this cytokine in protection against *E. caproni* infection. Interestingly, metacercariae administration to rIL-25-treated mice resulted in a reduction of endogenous IL-25 and IL-13 expression with respect to non-exposed rIL-25-treated mice, together with a transient boost of IL-12p35. This confirms that *E. caproni* infection in ICR mice does not activate IL-25 expression but, in contrast, induces IL-12 production which may explain the susceptibility to *E. caproni* primary infection.

Drug cure of primary infection resulted in a sudden decrease in cytokine expression with a concomitant overexpression of IL-25 and maintained tuft cell hyperplasia. Although it is difficult to explain the boost of IL-25 after worm elimination, this may well be related to the restoration of the intestinal epithelium, which is severely damaged because of the infection^16^. Previous studies showed that mechanisms for wound healing and epithelial restoration are activated early after *E. caproni* infection^33^,^34^. Our current results support that *E. caproni* primary infection activates several mechanisms to enhance epithelial homeostasis, in response to those processes that cause pathology. Although alternative activation of macrophages is common in intestinal helminth infections^35^,^36^, *E. caproni* primary infection caused overexpression of markers of both classical (IL-12 and iNOS) and alternative (ArgI and Ym-I) activation pathways. Similar to our results, *Citrobacter rodentium* infection in mice induced an intestinal Th1 environment with simultaneous classical and alternative activation of macrophages^37^. It was suggested that upregulation of ArgI was protective by enhancing the generation of polyamines in addition to competitive inhibition of iNOS. Modulation of the balance between iNOS and ArgI, and the arginase-ornithine decarboxylase metabolic pathway may represent a strategy for regulating intestinal inflammation^37^. Moreover, polyamines and Ym-I are useful for the repair of tissue damage^38^,^39^,^40^.

However, overproduction of polyamines may contribute to malignancy due to their cell proliferating effect and the oxidative stress caused by polyamine oxidase^37^. These features are consistent with the tissue alterations observed in responses to *E. caproni* infection in mice. *E. caproni* primary infection induces a pro-tumorigenic environment, with intestinal epithelial cell hyper-proliferation and increased epithelial restoration^33^,^34^. In this context, tuft cell hyperplasia and upregulation of IL-25 may participate in tissue repair after worm clearance. Th2 responses have been found to mediate the acute wound healing in intestinal helminth infections^41^. Moreover, tuft cells have been suggested to play a functional role, which may be critical in the epithelial restorative response to injury by activating, via IL-25, ILC2 to secrete IL-13 that acts on epithelial cell progenitors to promote their differentiation^28^,^42^. This boost of IL-25 induced by the cure of the primary infection may determine resistance against secondary infections. Challenge infection in an environment with elevated levels of IL-25 resulted in a significantly lower infection rate and worm recovery than in primary infections. Under these conditions, a Th2 phenotype with elevated levels of IL-4 and IL-13 and downregulation of IL-12p40 and IFN-γ was observed. Interestingly, the expression of IL-33 and TSLP was not affected after secondary infections, probably due to the lesser tissue damage induced^21^, which is supported by the low levels of iNOS. Interestingly, metacercariae challenge of resistant rIL-25-treated mice induced a peak of IL-12p35, though the presence of IL-25 prevented the development of a Th1 phenotype as it occurs in primary infections in naïve mice.

Protective type 2 responses against intestinal helminths have been associated with epithelial remodeling mediated by IL-13 and both quantitative and qualitative changes in intestinal mucus, among other mechanisms^35^,^43^,^44^. Our results indicate that partial and complete resistance to *E. caproni* infections are associated with goblet cell hyperplasia and changes in mucus glycosylation at the time of challenge infection. Although previous studies have suggested that goblet cell hyperplasia has little effect on resistance to *E. caproni* infections^16^,^45^,^46^, our current results are somewhat confusing. Primary infection induced a goblet cell hyperplasia of about 4-fold greater than in negative controls from 4 wppi. Cell counts remained elevated until the end of the experiment, concomitantly with the development of chronic infections and elevated worm recoveries. After pzq treatment, goblet cell levels returned to normal values, and secondary infection induced an earlier but lesser (about 2-fold) goblet cell expansion. Goblet cell hyperplasia appeared earlier in mice partially and completely resistant to infection. Although further evidence is needed, the early goblet cell hyperplasia observed in secondary infections and, mainly, in rIL-25-treated mice may contribute to resistance by hindering the establishment of newly excysted juvenile worms. In contrast, the later response that occurs in primary infections, may allow the development of juvenile to adult worms that could be resistant to the rejection-promoting effect of intestinal mucus.

As changes in mucin glycosylation induced by Th2 response have also been related to resistance to intestinal helminth infections, we investigated the specific staining of mucins in primary (chronic) infections, secondary (partially resistant) infections and resistant rIL-25-treated mice. Partial and complete resistance to infection were associated with elevated intensity of staining at the time of infection using WGA and UEA-I lectins. Previous studies have suggested that resistance to infection with the related species *Echinostoma trivolvis* is associated with increased expression of GlcNAcβ1,4 (GlcNAc) residues^47^. Moreover, elevated levels of GlcNAc and GlcNAcβ1,4 (GalNAc) GlcNAcβ1,4 residues were associated with resistance to superimposed *E. caproni* infections in rats infected with *Syphacia muris*^48^. Herein, we have observed that partial and complete resistance to *E. caproni* infection occurred when infection took place in an environment with elevated expression of GlcNAc residues as determined by staining with WGA. However, the results obtained using UEA-I lectin appear to be of more relevance. Complete resistance to infection in rIL-25-treated mice was associated with an early enhanced expression of terminal α-L-Fucosyl terminals (Fuc) residues in extracellular mucus and goblet cell vesicles, which is induced by IL-25 administration prior to infection. It has been shown that fucosylation of the small intestinal epithelium in response to infection may constitute a protective mechanism by enhancing host-microbial interactions during pathogen–induced stress^49^,^50^. Intensity of staining with UEA-I in secondarily infected mice was lower than in rIL25-treated mice which might be related to lesser quantity of IL-25 in the environment. Our results also suggest that exposure to *E. caproni* metacercariae regulates the expression of terminal Fuc residues to enhance parasite survival. Susceptibility of mice to primary infections was related with downregulation of terminal Fuc residues with respect to naïve mice. Moreover, exposure of rIL-25-treated mice to *E. caproni* metacercariae resulted in a marked reduction of the elevated expression of Fuc residues induced after rIL-25 treatment. Although the mechanism that determines the reduction of Fuc residues needs to be explored further, this change may serve to enhance parasite survival in absence of IL-25 in the milieu.

In summary, although the role of tuft cells and IL-25 in the outcome of intestinal helminth infections has been scarcely studied, our results indicate that they play a role in host resistance or susceptibility to infection. Susceptibility to primary infections is related to the inability of mice to express IL-25, despite expansion of tuft cells lineage. Under these conditions, a Th1 environment is developed, leading to chronic infections. In contrast, tuft cell hyperplasia induced by inoculation with exogenous IL-25 or due to epithelial restoration after cure of a primary infection enhanced tuft cell expansion and endogenous IL-25 production facilitating the development of protective Th2 responses with elevated levels of IL-13. Although the effector mechanisms responsible for the resistance need to be studied further, changes in goblet cell populations and increased expression of terminal Fuc residues in intestinal mucus appear to be involved in resistance against *E. caproni*.

## Material and Methods

### Parasites, hosts and experimental infections

The strain of *E. caproni* has been described previously^47^. Encysted metacercariae of *E. caproni* were removed from the kidney and pericardial cavity of experimentally infected *Biomphalaria glabrata* snails and used to infect male ICR mice weighing 30–35 g by gastric gavage (50 metacercariae each). The positivity of the infection was determined at necropsy or detection of eggs in stools as described previously^15^.

### Ethics Statement

This study has been approved by the Ethical Committee of Animal Welfare and Experimentation of the University of Valencia (Ref#A18348501775). Protocols adhered to Spanish (Real Decreto 53/2013) and European (2010/63/UE) regulations.

### Primary and secondary infections

A total of 90 mice were infected and randomly allocated to three groups (infected, pzq-treated and reinfected) of 30 mice each. The characteristics of the primary infections were studied in the animals in the infected group. These animals were maintained infected without additional treatment until the end of the experiment at 10 weeks post primary infection (wppi). At 4 wppi, animals in the other two groups, pzq-treated and reinfected, were treated with a double dose of 100 mg/Kg of praziquantel (pzq), orally administered on alternate days as described previously^21^. All mice belonging to these groups responded to the treatment and reverted to negative as determined by coprological examination. Two weeks after treatment, all mice in the reinfected group were challenged with 50 metacercariae of *E. caproni* following the same procedure used in primary infections. At 2, 4, 5, 6, 8, and 10 wppi, 5 animals of each group were necropsied to collect the materials required. The experiment was performed once, with 30 mice per group which were assayed as 5 replicates per each of 6 time points. Moreover, a total of 5 uninfected mice were used as controls at 0 wppi. The influence of the pharmacological treatment over the studied parameters was discarded since four additional mice were left uninfected, treated with pzq as described above and analyzed as the other animals. All the animals were maintained under conventional conditions with food and water *ad libitum*.

### Effect of IL-25 on *E. caproni* infection

To analyze the effect of IL-25 on the infection, a total of 40 mice were intraperitoneally injected with 0.5 μg of mouse recombinant IL-25 (rIL-25, R&D Systems) in saline buffer daily for 5 days and randomly allocated in two groups of 20 mice each: rIL-25-treated and exposed rIL-25-treated mice). The second day after starting with rIL-25 administration, animals in the exposed rIL-25-treated group were primarily infected with 50 metacercariae each, following the same procedure described above. Additionally, 5 mice were used as control and were injected with of saline buffer following the same protocol. At 3, 7 and 14 dppi, 5 the animals of each group were necropsied to determine the worm recovery and obtain the material required for the study.

### Total RNA extraction

Total RNA was extracted from full-thickness sections of ileum of necropsied mice. Total RNA was isolated using Real Total ARN Spin Plus kit (Durviz) according to the manufacturer’s instructions. The cDNA was synthesized using High Capacity cDNA Reverse Transcription kit (Applied Biosystems^®^).

### Real-Time PCR and relative quantification analysis

For quantitative PCR, 40 ng total RNA wasreverse transcribed to cDNA and added to 10 μL of TaqMan^®^ Universal PCR Master Mix, No AmpErase^®^ UNG (2x), 1 μL of the specified TaqMan^®^ Gene Expression Assay, and water to a final reaction volume of 20 μL. Reactions were performed on the Abi Prism 7000 (Applied Biosystems^®^), with the following thermal cycler conditions: initial setup of 10 min at 95 °C, and 40 cycles of 15 s denaturation at 95 °C and 1 min of annealing/extention at 60 °C each. Samples were amplified in a 96-well plate. In each plate, endogenous control, samples and negative controls were analyzed in triplicate. All TaqMan^®^ Gene Expression primers and probes for inducible nitric oxide synthase (iNOS), cytokines and mucins were designed by Applied Biosystems^®^ and offered as Inventoried Assays. The assay ID details are shown in Supplementary Table 1. Each assay contains two unlabeled primers and one 6-FAM™ dye-labeled, TaqMan^®^ MGB probe. Primer concentration was optimized by a matrix of reactions testing a range of concentrations for each primer against different concentrations of the partner primer and also negative controls were included.

Cycle threshold (Ct) value was calculated for each sample, housekeeping and uninfected control. To normalize for differences in efficiency of sample extraction or cDNA synthesis we used β-actin as housekeeping gene. To estimate the influence of infection in the expression levels we used a comparative quantification method (2^−ΔΔCT^ – method)^51^. This method is based on the fact that the difference in threshold cycles (ΔCt) between the gene of interest and the housekeeping gene is proportional to the relative expression level of the gene of interest. The fold change in the target gene was normalized to β-actin and standardized to the expression at time 0 (uninfected animals) to generate a relative quantification of the expression levels^52^.

### Analysis of goblet cell responses

Goblet cell responses to *E. caproni* infections in the ileum of mice were evaluated in primary and secondary infections and in rIL-25-treated mice. At each time point, 5 mice in each group were necropsied and ileal sections of about 0.7 cm in length were obtained and fixed in 4% paraformaldehyde (PFA). After embedding in paraffin wax, serial 4 μm-sections were cut from each tissue block and stained with alcian blue. Due to the severe tissue damage induced by primary *E. caproni* infection, which makes it difficult to count the number of cells per villus-crypt unit, results are expressed in number of cells per high power field (HPF) (400x), studied over 10 selected HPFs. Results are expressed as the mean number of cells per HPF ± standard deviation.

### Analysis of tuft cell responses

Tuft cell populations were study using anti-doublecortin-like kinase 1 (Dclk1) antibody, a molecule that serve as a marker of these cells. Fluorescent immunohistochemistry was performed on paraffin-embedded tissue sections, basically as described previously^46^. Goat antibody anti-Dclk1 (ab31704, Abcam) was used as primary antibody for staining intestinal tuft cells^27^. Anti-Dclk1 was diluted 1/1,000 in PBS containing 0.3% Triton™ X-100 and 10% FCS and incubated for 18 h in a humid chamber at room temperature, under continuous agitation. After washing 3 times in PBS, intestinal sections were incubated for 2 h with secondary antibody, goat anti-rabbit IgG conjugated with Alexa Fluor^®^ 647 (Jackson ImmunoResearch Laboratories, Inc.), diluted 1/100 in PBS-Triton™ X-100 (0.3%). Slides were washed in PBS and cell nuclei were counterstained with DAPI before mounting with Fluoromount™ (Sigma-Aldrich). Cell staining was analyzed by fluorescence microscopy. Results are expressed as the mean number of cells per field (200x) ± standard deviation, studied over 5 selected fields.

### Lectin histochemistry and confocal microscopy

Glycosylation patterns in the ileum of *E. caproni*-infected mice were determined using a lectin-biotin, avidin-peroxidase method on PFA-fixed sections of ileal samples, as described previously^46^. Briefly, tissue sections were deparaffinized and rehydrated. A blocking solution of 5% BSA (Roche) and 0.2% Triton™ X-100 (Sigma-Aldrich) in PBS was applied for 1 h. After washing, slides were incubated for 1.5 h with a panel of 4 different biotinylated lectins: *Dolichos biflorus* agglutinin, DBA; *Ulex europeaus* agglutinin, UEA-I; *Triticum vulgaris* agglutinin, WGA; and *Sambucus nigra* agglutinin, SNA (all of them from Sigma-Aldrich) at proper dilutions in PBS, containing Triton™ X-100 (0.2%). After washing, the sections were incubated with the avidin-peroxidase conjugate diluted 1:1,000 in PBS with Triton™ X-100 (0.2%) for 1 h, followed by repeated washes. Next, the slides were incubated with goat antibody anti-peroxidase, conjugated with fluorescein isothiocyanate (FITC) (Jackson InmunoResearch) at a dilution of 1:200 in PBS for 1 h in darkness. Finally, the sections were counterstained with DAPI and mounted using the Fluoromount™ procedure. In order to determine potential autofluorescence or unspecific binding, negative controls were used. They were processed as described above with the exception of the incubation with lectins. The histological staining was evaluated as described previously^53^ and was ranked using (−) for absent, (+) for just detectable (++) for positive and (+++) for strongly positive. The slides were analyzed by multitracking on a FV10I confocal microscope (Olympus). A total of 5 intestinal sections were analyzed for each animal at each time point. The assessment of the histological staining was carried out blindly.

### Statistical analysis

χ^2^ test was used to compare the rates of infection between both groups of mice at each week post-infection. To compare the worm recovery between primary and challenge infections, a Student’s t-test was used at each week post-infection. One-way ANOVA with Bonferroni test as post-hoc analysis were used to compare expression levels of cytokines, enzymes, goblet cells and tuft cells at each time point. P < 0.05 was considered as significant. Prior to analyses, data were log transformed to achieve normality and verified by the Anderson–Darling Test.

## Additional Information

**How to cite this article**: Muñoz-Antoli, C. *et al*. Interleukin-25 Induces Resistance Against Intestinal Trematodes. *Sci. Rep.* **6**, 34142; doi: 10.1038/srep34142 (2016).

## Supplementary Material

Supplementary Information

## Figures and Tables

**Table 1:** Lectin staining, subjectively described from +++ (very strong) to − (absent) in infected, pzq-treated and reinfected mice at different weeks post-primary infection (wppi), compared with non-infected controls (0 wppi).

	Groups of mice	wppi	Lectin staining			
			Brush border	Mucus	Goblet cell vesicles	
					Staining	Predominance
WGA	Control	0	−	−	−	NA
	Infected	2	+	−	+/−	−
		4	+	+/−	+	
	Pzq-treated	5	++	++	++	NA
		6	++	++	++	NA
	Reinfected	8	++	++	++	NA
		10	+++	+++	+++	NA
DBA	Control	0	++	++	++	NA
	Infected	2	++	++	++	NA
		4	+++	+++	+++	NA
	Pzq-treated	5	+	+/−	+/−	+
		6	++	++	++/−	++
	Reinfected	8	++	+	+/−	−
		10	+++	+++	+++	NA
UEA-I	Control	0	−	++	+	NA
	Infected	2	+/−	++	+++	−
		4	+/−	++	++	NA
	Pzq-treated	5	−	+/−	+/−	+
		6	−	+/−	+/−	+
	Reinfected	8	−	+	+/−	+
		10	−	+	+/−	+
SNA	Control	0	−	+	+/−	−
	Infected	2	+/−	+	+/−	−
		4	+/−	+	+	−
	Pzq-treated	5	−	+	+/−	−
		6	−	++	+/−	+
	Reinfected	8	−	++	++/−	+
		10	−	++	+/−	

The staining was independently evaluated in brush border, mucus and goblet cell vesicles. In case that both stained and non-stained vesicles were observed, the predominant ones are indicated in the right column. When all vesicles showed the same staining pattern this distinction is not applicable (NA). Glycans recognized by each lectin are GlcNAcβ1,4 (WGA), GalNAcα1,3(LFucα1,2)Galβ1,3/4GalNAcβ1- (DBA), α-L-Fucosyl terminals (UEA-I).

**Table 2:** Lectin staining, subjectively described from +++ (very strong) to − (absent) in rIL-25-treated and rIL-25-treated and infected mice with *Echinostoma caproni* metacercariae.

Lectin	rIL-25 treated mice				Exposed rIL-25 treated mice				
	dpt (dpi)	Brush border	Mucus	Goblet cell vesicles		Brush border	Mucus	Goblet cell vesicles	
				Staining	Predominance			Staining	Predominance
WGA	0 (0)	−	−	−	NA	−	−	−	NA
	2 (0)	+/−	+	+/−	+	+/−	+	+/−	+
	5 (3)	+/−	++	++/−	++	+	+	+/−	+
	9 (7)	++/−	+++	++/−	++	+/−	+	+/−	−
	16 (14)	+/−	++	++/−	++	+/−	+/−	+/−	−
DBA	0 (0)	++	++	++	NA	++	++	++	NA
	2 (0)	++	++	++/−	++	++	++	++/−	+
	5 (3)	++	++	++/−	++	++	++	++	NA
	9 (7)	+++	+++	+++	NA	++	+++	++	NA
	16 (14)	++	++	++/−	++	+	+	+/−	+
UEA-I	0 (0)	−	++	+	NA	−	++	+	NA
	2 (0)	−	+++	+++/−	+++	−	+++	+++/−	+++
	5 (3)	−	+++	+++/−	+++	−	++/−	++/−	++
	9 (7)	−	+++	+++/−	+++	+/−	+	+	NA
	16 (14)	−	+++	+++/−	+++	−	+	+/−	−
SNA	0 (0)	−	+	+/−	−	−	+	+/−	−
	2 (0)	−	+	+/−	−	−	+	+/−	−
	5 (3)	−	++/−	++/−	++	−	+/−	+/−	+/−
	9 (7)	−	++/−	++/−	++	−	++/−	++/−	++
	16 (14)	−	+	+/−	−	−	+/−	+/−	−

The staining was independently evaluated in brush border, mucus and goblet cell vesicles. In case that both stained and non-stained vesicles were observed, the predominant ones are indicated in the right column. When all vesicles showed the same staining pattern this distinction is not applicable (NA). dpt: day after starting with intraperitoneal administration of IL-25; dppi: day after challenge with *Echinostoma caproni* metacercariae.
