# Supplementary material for: Interleukin-25 Induces Resistance Against Intestinal Trematodes
Source: Sci Rep. 2016 Sep 23;6:34142. doi: 10.1038/srep34142 (PMC5034261; doi:10.1038/srep34142)
Supplement: Supplementary Information [file srep34142-s1.pdf]

Interleukin-25 Induces Resistance Against Intestinal Trematodes

Carla Muñoz-Antoli, Alba Cortés, Rebeca Santano, Javier Sotillo, J. Guillermo Esteban, Rafael Toledo

Supplementary Figure 1. Production of several cytokines was not affected in primary and secondary infections. Expression of cytokine mRNA in the intestinal tissue of ICR mice infected, praziquantel (pzq)-treated and reinfected with *Echinostoma caproni* for which no significant changes were detected. The relative quantities (RQ) of cytokine genes are shown after normalization with β-actin and standardization of the relative amount against day 0 sample. Vertical bars represent the standard deviation. a: significant differences with respect to negative controls; b: significant differences between groups at each week of the study ($p<0.05$).

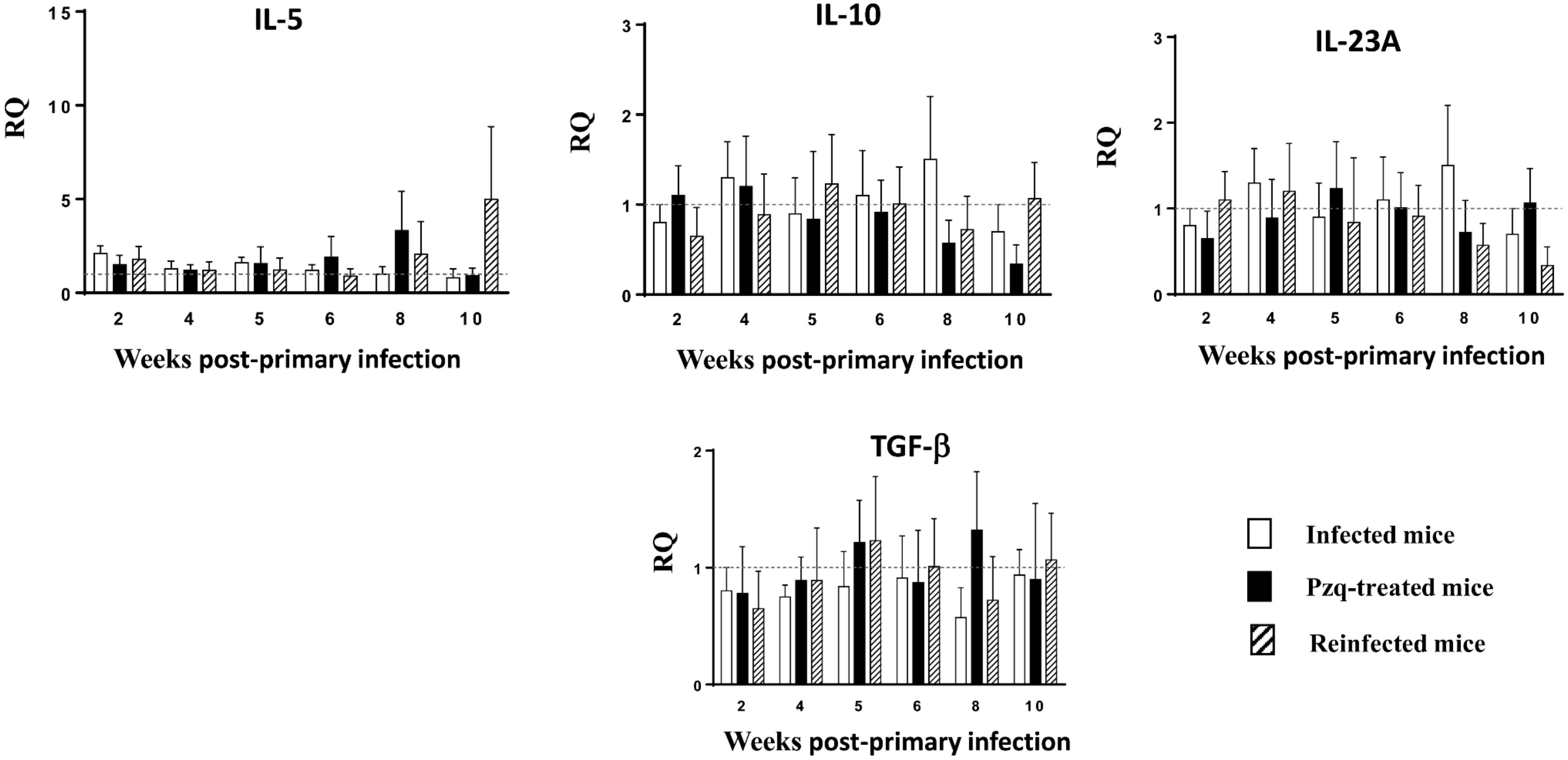
IL-5
IL-10
IL-23A
TGF-β
RQ
Weeks post-primary infection
2
4
5
6
8
10
Infected mice
Pzq-treated mice
Reinfected mice

9

Table S1 Applied Biosystems Inventoried assays used

Assay ID Details	
	Mice
β-actin	Mm01205647_g1
IL-2	Mm00434256_m1
IL-4	Mm00445259_m1
IL-5	Mm99999063_m1
IL-6	Mm00446190_m1
IL-9	Mm00434305_m1
IL-10	Mm00439614_m1
IL-12p35	Mn00434165_m1
IL-12p40	Mn00434174_m1
IL-13	Mm99999190_m1
IL-17	Mm00439618_m1
IL-22	Mm01226722_g1

IL-23A	Mn00518984_m1
IL-25	Mm00499822_m1
IL-33	Mm00505403_m1
IFN-γ	Mm99999071_m1
TGF-β	Mn001178820_m1
TNF-α	Mm99999068_m1
TSLP	Mm01157588_m1
Arg I	Mm00475988_m1
Arg II	Mm00477592_m1
Ym-I	Mm00657889_mH
iNOS	Mm01309897_m1
